# Modified genome comparison method: a new approach for identification of specific targets in molecular diagnostic tests using *Mycobacterium tuberculosis* complex as an example

**DOI:** 10.1186/s12879-018-3417-x

**Published:** 2018-10-12

**Authors:** Alireza Neshani, Reza Kamali Kakhki, Mojtaba Sankian, Hosna Zare, Amin Hooshyar Chichaklu, Mahsa Sayyadi, Kiarash Ghazvini

**Affiliations:** 1 Antimicrobial Resistance Research Center, Mashhad University of Medical Sciences, Mashhad, Iran; 2 Student Research Committee, Mashhad University of Medical Sciences, Mashhad, Iran; 3 Immunology Research Center, School of Medicine, Mashhad University of Medical Sciences, Mashhad, Iran; 4 Department of Microbiology and Virology, School of Medicine, Mashhad University of Medical Sciences, Mashhad, Iran

**Keywords:** *Mycobacterium tuberculosis* complex, PCR, Genome comparison method, Specific target, 5KST

## Abstract

**Background:**

The first step of designing any genome-based molecular diagnostic test is to find a specific target sequence. The modified genome comparison method is one of the easiest and most comprehensive ways to achieve this goal. In this study, we aimed to explain this method with the example of *Mycobacterium tuberculosis* complex and investigate its efficacy in a diagnostic test.

**Methods:**

A specific target was identified using modified genome comparison method and an in-house PCR test was designed. To determine the analytical sensitivity and specificity, 10 standard specimens were used. Also, 230 specimens were used to determine the clinical sensitivity and specificity.

**Results:**

The identity and query cover of our new diagnostic target (5KST) were ≥ 90% with *M. tuberculosis* complex. The 5KST-PCR sensitivity was 100% for smear-positive, culture-positive and 85.7% for smear-negative, culture-positive specimens. All of 100 smear-negative, culture-negative specimens were negative in 5KST-PCR (100% clinical specificity). Analytical sensitivity of 5KST-PCR was approximately 1 copy of genomic DNA per microliter.

**Conclusions:**

Modified genome comparison method is a confident way to find specific targets for use in diagnostic tests. Accordingly, the 5KST-PCR designed in this study has high sensitivity and specificity and can be replaced for conventional TB PCR tests.

**Electronic supplementary material:**

The online version of this article (10.1186/s12879-018-3417-x) contains supplementary material, which is available to authorized users.

## Background

One of the methods to find a specific target to use in diagnostic tests is genome comparison method. In this method, the full genome of the organism is compared with close organisms and the most specific sequences can be identified and used for the design of nucleic acid-based diagnostic tests [1]. Unfortunately, this method causes confusion due to the lack of transparency in the protocol, and use of the unfamiliar software. In this study, we applied some changes to the previous method such as defining an obvious pathway using online and available software and aimed to employ this method to find a new specific target for *Mycobacterium tuberculosis* complex. It is clear that this new approach to detect specific diagnostic targets is also appropriate in other microorganisms, and it makes the first step of designing any genome-based diagnostic test (finding a specific target) easier.

Tuberculosis (TB) has been among the 10 leading causes of human death since 2000 [2]. It causes more than 1.3 million deaths each year, which is even higher than road traffic accidents and HIV/AIDS [2].

WHO has planned for a 90% reduction in TB deaths by 2030. To achieve this, both parts of diagnosis and treatment must be strengthened. TB is a treatable infection, provided it is diagnosed promptly. The importance of the diagnostic part becomes clearer by knowing this fact that timely diagnosis and treatment from 2000 to 2016, saved the lives of over 53 million people with TB [2].

So far, many laboratory tests have been designed to diagnose TB, but some of these tests despite their high levels of performance, have not been used in high TB burden countries due to the costs [3]. The most common methods for TB diagnosis in low-income countries include acid-fast bacilli microscopy, culture and PCR [4].

PCR is the most important molecular diagnostic method used to detect TB during past two decades, and it has maintained its position despite the introduction of newer methods such as *loop*-mediated isothermal amplification (LAMP). Over the past years, various target sequences were used for TB PCR such as *IS6110*, *mpb64*, *devR*, *hsp65*, 38 KDa (*pstS1*), 30 KDa (*fbpB*), *esat6*, *cfp10*, 16S rDNA gene (*rrs*) and *rpoB* [5–9]. Diagnostic tests based on these targets have high sensitivity and specificity. Nevertheless, sensitivity of the test is greatly reduced on clinical specimens [10, 11]. This reduction of sensitivity can be attributed to the presence of inhibitors in clinical specimens. According to the results of some articles, there are unknown substances in the respiratory specimens (especially sputum) that have been shown to cause 10 to 26% false-negative results in PCR test and thereby reduce the sensitivity [12–14]. other items such as poorly designed primers [15], or partial loss of target DNA during purification [16] are also effective in the reduction of PCR sensitivity, especially on clinical specimens.

Each of the targets used to date has limitations. For example, about two widely used targets (*IS6110*, *mpb64*), it has been shown that in some parts of Southeast Asia, there are strains of *M. tuberculosis* which lack the *IS6110* sequence [17], and the nonspecifically positive result of *mpb64*-PCR was also seen in *M. scrofulaceum* [18].

Therefore, we still need to find a specific and long enough sequence that would allow us to design the best possible diagnostic primers with minimal mispairing, hairpin, and dimer, to enhance the PCR sensitivity.

In this study, by modifying the genome comparison method, we identified a 5 Kbp sequence which is specific for *M. tuberculosis* complex (MTBC) and named it 5KST (5 Kbp Specific Target). It was then used to design a sensitive and highly specific PCR test. Next, the efficacy of this target was evaluated on clinical specimens (true positives and negatives) and pure genomic DNA.

## Methods

Employment of this method for the genome of *Mycobacterium tuberculosis* with about 4.4 million bp, required 45–50 h of continuous work. To reduce the errors, all dedicated work of finding the target was divided between 4 people and each part was performed separately. With this approach, 11–12 h were needed for them to investigate their fragments, which were included in three working days (4 h of continuous work per day).

### Finding specific target by modified genome comparison method

**Fig. 1:**
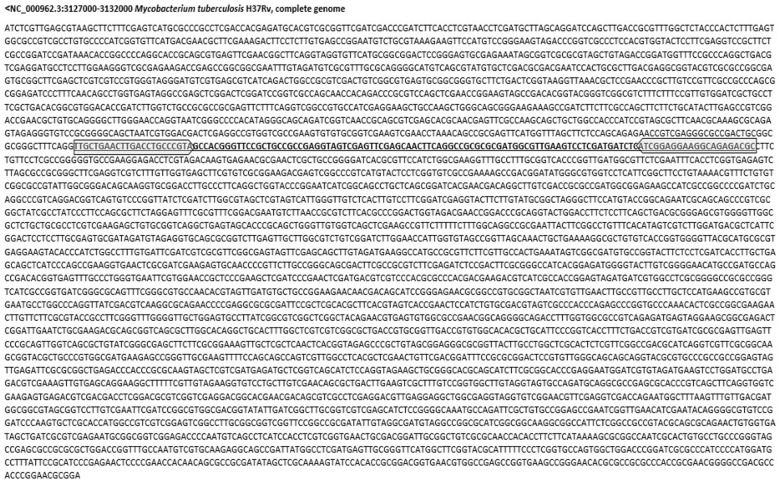
The sequence of 5 Kb Specific Target (5KST). The 5KST primers are shown

At this stage, the complete genome sequences of MTBC members were compared with available genomic sequences on nucleotide collection database (a part of NCBI) and the most specific sequence was selected. It should be noted that this instruction can be accomplished for any other microorganisms. The steps are summarized as follows:

1. First, available genomic sequences of MTBC members on NCBI database were determined and one case, preferably RefSeq, was considered as the reference for each species (Table 1).
2. Then, one of these seven items should be selected as the initial basis. For this study, the sequence of *M. tuberculosis* H37Rv (NC_000962.3) was considered as the basis and its genomic sequence was downloaded from the NCBI and cut to about 5000 bp fragments, providing about 882 fragments.
3. Each fragment was compared separately with other NCBI available genomic sequences by blastn (https://blast.ncbi.nlm.nih.gov/Blast.cgi). The required time for each fragment was about 30 s.
4. Then, the blast search results were evaluated, and appropriate fragments were selected. Two criteria were considered for the result evaluation: 1. Presence of all 7 species of MTBC (Table 1) in Blast search results, with both identity and query cover ≥90%. To simplify, we used Ctrl+F of windows operating system. 2. No bacteria other than MTBC members would appear with query cover > 10%. Blast search results are sorted by their query cover (by default) and are shown in descending order. So, the bacteria with a query cover > 10% can be easily found with a glance at the end of the list. The required average time for this step was about 2 min to check each fragment.
5. For the second screening, from selected sequences of previous step, the sequence which could identify the most of MTBC members was considered as the most specific sequence (Fig. 1).

**Table 1:** MTBC members with available complete genome on NCBI database (preferably RefSeq)

Species	Accession number
*Mycobacterium tuberculosis* H37Rv	NC_000962.3
*Mycobacterium africanum* GM041182	NC_015758.1
*Mycobacterium bovis* AF2122/97	NC_002945.4
*Mycobacterium bovis* BCG Pasteur 1173P2	NC_008769.1
*Mycobacterium canettii* CIPT 140010059	NC_015848.1
*Mycobacterium caprae* strain Allgaeu	NZ_CP016401.1
*Mycobacterium microti* strain 12	CP010333.1

### Bioinformatics control

A) Comparison with *rpoB*: In this part the *rpoB* gene, an essential target sequence, was used as a positive control in bioinformatic investigations and *rpoB* sequence compared with all registered complete genomes in the nucleotide collection database (*https://www.ncbi.nlm.nih.gov/nuccore/?term=*), using blastn search. Then, all strains of *M. tuberculosis* (with complete genome) identified by this target, were extracted and compared with the strains identified by 5KST. The identity of these two groups showed that the 5KST is conserved among the all registered *M. tuberculosis* strains in nucleotide collection database.

B) Investigating the 5KST conservation with the help of *TB-ARC project* data:

The genomic sequence of 304 strains of *M. tuberculosis*, *M. africanum*, and *M. bovis* related to the TB Antibiotic Resistance Catalog project [19] around the world, were randomly downloaded from the site *(**http://olive.broadinstitute.org/projects/tb_arc**)* and the presence of 5KST sequence among these 304 strains was studied by blastn search. The geographic regions and the number of randomly studied strains for each region are listed in Table 2.

**Table 2:** The number of randomly studied strains and their geographical regions, to prove the 5KST conservation

Location	Number of strains
India	53
Sweden	36
Iran	8
Uganda and South Korea	40
Moldova	16
South Africa(KwaZulu-Natal)	56
Africa	51
Mali	27
Romania	9
USA	8
Total	304

### Primer design

Designing and analysis of primers for the 5 Kbp Specific Target (5KST) was performed with Oligo7 [20] and Oligoanalyzer 3.1 software (https://eu.idtdna.com/calc/analyzer). Primers and 128 bp amplicon sequence are shown in Table 3.

**Table 3:** Primers and the amplicon sequence of 5KST-PCR

Forward	5’-TTGCTGAACTTGACCTGCCCGTA
Reverse	5’-GCGTCTCTGCCTTCCTCCGAT
Amplicon	5’TTGCTGAACTTGACCTGCCCGTAGCCACGGGTTCCGCTGCCGCCGAGGTAGTCGAGTTCGAGCAACTTCAGGCCGCGCGCGATGGCGTTGAAGTCCTCGATGATCTCATCGGAGGAAGGCAGAGACGC

### Polymerase chain reaction

PCR reaction was prepared in the total volume of 25 μl containing 1 μl of sample DNA, 2.5 μL of 10× PCR buffer (100 mM Tris-HCl [pH 8.3], 500 mM KCl), 1.5 μL of 25 mM MgCl_2_, 0.5 μL of 200 μM (each) of the four dNTPs, 1 μL of each 10 μM forward and reverse primers, PCR grade water, and 0.625 U of *Taq DNA polymerase*. The positive control contained 1 ng of *M. tuberculosis* purified DNA, and the negative control contained no DNA.

The DNA amplification was performed by thermocycler (Atlas G Japan). Initial denaturation at 95 °C for 5 min, was proceeded by 40 cycles of (i) denaturation at 95 °C for 15 s, (ii) annealing at 60 °C for 20 s, (iii) extension at 72 °C for 30 s, followed by a final extension at 72 °C for 5 min.

Then, 5 μL of PCR product was electrophoresed on 2% agarose gel containing DNA green viewer and DNA bands were visualized under ultraviolet light from UV transilluminator (UVTEC). The presence of a 128 bp fragment indicated the positive result. 10% of positive specimens were selected randomly and sequencing was performed on their amplicons.

### Analytical sensitivity (detection limit)

Purified genomic DNA of *M. tuberculosis* H37Rv was extracted from colonies by Dick Van Soolingen method [21]. DNA concentration was determined by spectrophotometer (Thermo Scientific). Then, serial dilution of genomic DNA was prepared using distilled water (10 pg, 1 pg, 100 fg, 10 fg, 5 fg, 1 fg) and 1 μL was used as template. This process was performed for three times, on three different days.

### Analytical specificity

To determine the analytical specificity for 5KST-PCR, genomic DNA of two members of MTBC (*M. tuberculosis* H37Rv and *M. bovis* BCG) and four Non-tuberculosis mycobacteria (*M. smegmatis*, *M. chelonae*, *M. simiae*, *M. fortuitum*) and four non-Mycobacterium bacteria (*Corynebacterium diphteriae*, *Escherichia coli*, *Staphylococcus aureus* and *Streptococcus pneumoniae*) were used which purchased from Pasteur Institute (Tehran, Iran). The amount of 10 ng of genomic DNA was used in the reaction for each species.

### Evaluation of clinical specimens

#### Specimen collection

A total of 100 smear-positive and culture-positive, and 100 smear-negative and culture-negative sputum specimens were obtained from tuberculosis laboratory of Qaem hospital in Mashhad. The ethical approval for performing this study was obtained from the Ethics Committee of the Mashhad University of Medical Sciences (Ethics code of IR.mums.fm.rec.1397.143). To confirm TB, *mpb64*-PCR and *IS6110*-PCR were performed on colonies of each specimen [22].

#### Specimen processing

To homogenize and concentrate the specimens, all clinical specimens examined in this study was processed using modified Petroff’s method and cultured on LJ medium [23]. In brief, 5 ml of sputum was homogenized for 15 min in a shaker using an equal volume of 4% NaOH. After centrifugation at 6000 rpm for 15 min, the sediment was neutralized with 20 ml of sterile distilled water. The samples were again centrifuged. The supernatant was discarded and the precipitate was dissolved in the little residual liquid in the bottom of falcon to be used for the next steps.

#### Providing smear negative, culture-positive specimens

A total of 30 smear-positive and culture-positive processed samples were diluted with the processed negative specimen and two-fold serial dilutions were prepared (1/2, 1/4, 1/8, 1/16, 1/32, 1/64, 1/128). Then, all of the dilutions were subjected to ziehl-neelsen staining and smear-negative dilutions were cultured on Lowenstein Jensen medium (LJ). The lowest concentrations with a positive culture were considered as smear-negative, culture-positive specimens.

#### DNA extraction

Autoclave method with some modification was used to extract DNA [24]. The processed specimens were transferred to 1.5 ml microtubes. Then, microtube lids were sealed and the autoclave process was performed (121°c for 5 min). No other substance was added to the specimens. In order to remove the debris, microtubes were centrifuged at 12000 rpm for 5 min. Then, the supernatant was used as the template for 5KST-PCR test.

#### Investigating the inhibitory effect of clinical specimens on 5KST-PCR

To study the inhibitory effect of clinical specimens, 8 true negative processed sputum samples were pooled together. Then the pure genomic DNA provided in the previous step, was added to 50 μl of this pooled specimen up to the final concentration of 100 pg/μl of DNA. Then, two groups of six dilutions were prepared by the remained pooled specimen. The first group included 10 pg, 1 pg, 100 fg, 10 fg, 5 fg, and 1 fg dilutions and the second group included 5 pg, 0.5 pg, 50 fg, 5 fg, 2.5 fg, and 0.5 fg dilutions. Then, 1 μl of DNA for the first group and 2 μl of DNA for the second group were used for the 5KST-PCR test. So, the amount of DNA used in both groups was equal and the only difference was the amount of clinical specimen used in the reactions.

## Results

### Target finding

The 5000 bp fragment containing nucleotides 3,127,000 to 3,132,000 of *M. tuberculosis* H37Rv genome (NC_000962.3) was identified as the most specific possible sequence (Fig. 1). The blastn search showed that among the NCBI-registered complete genomes (nucleotide collection database), this sequence could specifically detect 237 strains of *M. tuberculosis* (Additional file 1: Table S1), 12 strains of *M. bovis* BCG, 8 strains of *M. bovis*, 3 strains of *M. africanum*, 2 strains of *M. canettii*, 1 strain of *M. caprae* and 1 strain of *M. microti*.

### Results of bioinformatics control

Two hundred thirty-seven strains of *M. tuberculosis* (with complete genome registered in nucleotide collection database) could be identified by *rpoB* sequence, which was quite identical to the strains which could be identified by 5KST-PCR (Additional file 1: Table S1).

Furthermore, the study of 5KST presence in 304 strains of *M. tuberculosis* related to the TB-ARC project also showed that this sequence is present in the all 304 strains and is considered as a conserved sequence (Additional file 1: Table S2).

### Analytical sensitivity (detection limit)

**Fig. 2:**
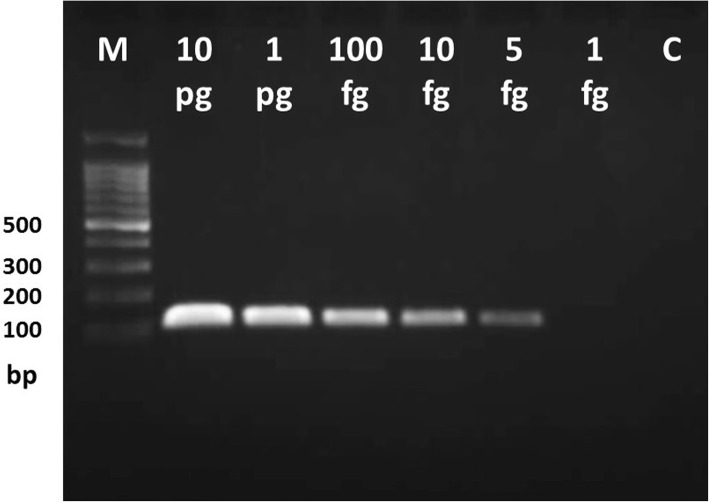
Gel electrophoresis of 5KST-PCR products. It shows target amplicons at different concentrations of M. tuberculosis H37Rv genomic DNA as template. Lane M shows 100 bp DNA ladder and successive lanes show amplicons using 10 pg, 1 pg, 100 fg, 10 fg, 5 fg, 1 fg of M. tuberculosis genomic DNA. Lane C shows negative control

Analytical sensitivity is the lowest DNA concentration that a test can detect. Accordingly, the 5KST-PCR analytical sensitivity was 5 fg approximately equivalent to 1 copy of *M. tuberculosis* genomic DNA per μl (Fig. 2).

### Analytical specificity

Both species of *M. tuberculosis* and *M. bovis* BCG which studied in this study were identified by 5KST-PCR, but no amplicon was produced for studied Non-tuberculosis mycobacteria (*M*. *smegmatis*, *M. chelonae*, *M. simiae*, *M. fortuitum*) and non-Mycobacterium bacteria (*Corynebacterium diphteriae*, *Escherichia coli*, *Staphylococcus aureus* and *Streptococcus pneumoniae*) (Table 4).

**Table 4:** The results for analytical specificity of 5KST-PCR

Groups	Bacterial Species	5KST-PCR result
*M. tuberculosis complex*	*M. tuberculosis* H37Rv	**+**
	*M. bovis* BCG	**+**
Non-tuberculosis mycobacteria	*M*. *smegmatis*	**-**
	*M. chelonae*	**-**
	*M. simiae*	**-**
	*M. fortuitum*	**-**
Non-Mycobacterium bacteria	*Corynebacterium diphteriae*	**-**
	*Escherichia coli*	**-**
	*Staphylococcus aureus*	**-**
	*Streptococcus pneumoniae*	**-**

### Clinical specimen’s result

Before beginning the study, all the specimens were cultured on the LJ medium. To select the true positive specimens, DNA extraction of the colonies was performed by simple boiling method and positive specimens were confirmed by *mpb64*-PCR and *IS6110*-PCR.

Only those specimens were included in the study as the true positives, which had clinical symptoms of the patient, and also simultaneous positive results of acid-fast bacilli smear, culture, and both PCR tests on the colonies. In addition, only those negative specimens were included in the study as true negatives, which had negative result of acid-fast bacilli smear and the culture, with the final clinical diagnosis of non-tuberculosis infection.

#### Clinical sensitivity and specificity

All 100 smear-positive, culture-positive specimens were positive for 5KST-PCR test (100% sensitivity) and 25 of 30 smear-negative, culture-positive samples were positive by the test (85.7% sensitivity). All 100 smear-negative and culture-negative specimens, were negative in 5KST-PCR test (100% clinical specificity). The 5KST-PCR results on clinical specimens are provided in Table 5.

**Table 5:** 5KST-PCR results on clinical specimens

	Clinical sensitivity (Smear +,culture +)	Clinical sensitivity (Smear -,culture +)	Clinical specificity (Smear -,culture -)
5KST-PCR result	100%	85.7%	100%

#### Inhibitory effect of clinical specimens on 5KST-PCR

The results showed the sensitivity of 5 fg/μl and 10 fg/μl when using 1 μl and 2 μl of processed clinical specimen in our test, respectively. (Additional file 1: Figure S1).

## Discussion

One of the most important parts of designing a nucleic acid amplification test (NAAT) such as PCR, is having a completely specific and long enough target. Specificity of the target would eliminate the false positive results. The long sequence would help the researcher to design efficient and sensitive primers. In this study, genome comparison method with few modifications was used to achieve completely specific and long sequence (1). In this method, using bioinformatic facilities provided by the NCBI database, genomic sequences of 7 members of MTBC were compared with complete genomes available on the database (up to 2018.08.20) and a 5000 bp sequence with high specificity was identified.

Based on in silico studies, the 5KST can specifically detect the most of NCBI-registered complete genomes of MTBC members including: 237 strains of *M. tuberculosis*, 12 strains of *M. bovis* BCG, 8 strains of *M. bovis*, 3 strains of *M. africanum*, 2 strains of *M. canettii*, 1 strain of *M. caprae* and 1 strain of *M. microti*.

Furthermore, the investigation of 5KST presence in 304 strains of *M. tuberculosis* from different parts of the world (TB-ARC project) showed that this sequence is conserved among all these strains (Additional file 1: Table S2). It should be noted that few of these 304 strains had the query cover of less than 100% (about 81–85%). After evaluating the sequences of these strains, we found that the query cover of less than 100% was not due to the less similarity to our sequence, but also it resulted from the gaps of the sequence during the sequencing process that recorded NNN’s instead of G, C, A, T bases.

Tuberculosis is a disease which has caused millions of deaths so far. Despite the advances in human science, it still puts millions of people at the risk of death every year. Therefore, extensive studies are being conducted in many parts of the world to control it in prevention, diagnosis and treatment parts [2]. The aim of the present study was to improve the diagnostic part of TB. If the diagnosis is timely, TB can be controlled and treated with minimal side effects when the disease is not advanced [25].

Three tests that are routinely used in most TB laboratories include acid-fast bacilli microscopy, culture and PCR [26]. PCR is fast, sensitive and highly specific. In most studies on clinical specimens, it showed higher sensitivity than acid-fast bacilli microscopy, but lower or equal sensitivity than culture. PCR acts more specific than the two other tests [26–28].

The most important factors that reduce the sensitivity of the PCR test on clinical specimens include 1. Presence of inhibitors in clinical specimens 2. Partial loss of DNA during purification 3. Chemical inhibitors residue from the purification process 4. Poorly designed and inefficient primers [16, 28, 29].

In this study, to prevent the negative effects of inhibitors in clinical specimens, only 1 μL of the specimen was used in 25 μL of reaction. The results of this study, previous experiences of our team in molecular laboratory, as well as reviewing the studies of other researchers, showed that the sensitivity of PCR test is lower when using clinical specimens (either natural or artificial) than pure DNA in the reaction. We don’t know exactly which inhibitor exists, but generally the substances of clinical specimens, especially sputum, cause the reduction in the sensitivity of test. As JE Clarridge et al. (1993), in a large study to evaluate the PCR test on clinical specimens, showed that the substances of clinical specimens, especially sputum, could cause up to 20% false-negative results and reduce the sensitivity [14]. In another study on sputum specimens, FS Nolte et al. reported that the sputum could produce 10 to 17% false-negative [12]. In a study on 76 respiratory specimens in Turkey, the inhibition rate of up to 26% was observed in the real-time PCR test and caused false-negative results [13]. Also in our study, the use of ≥2 μl clinical specimen in the 25 μl 5KST-PCR reaction worsened the sensitivity from 5 fg to 10 fg.

Also, to prevent DNA loss during purification, this step was removed and only autoclave extraction with few modifications was performed. Furthermore, since the 5KST sequence is long enough, we were able to design efficient primers with the best probable condition of not producing hairpin and dimer structures.

Our studies with blastn showed that all the important targets that have been used so far, have short length or contain long nonspecific regions. Unlike other targets, the 5KST target in addition to being long enough (5000 bp), does not have any statistically significant relationship to NTM bacteria. Some of the targets that have been used more than others or had better detection limit include: *rpoB*, *IS6110*, *devR*, *mpb64*, *sdaA*.

*IS6110* has been used as one of the most widely used diagnostic targets for TB [8]. This 1361 bp sequence, is repeated multiple times in the genome of *M. tuberculosis* and thus is regarded as a sensitive diagnostic target. Up to now, various PCR tests are designed based on this sequence, and the reported detection limit usually equals 2 copy of *M. tuberculosis* genomic DNA per μl [18, 30–32]. Various studies showed that the PCR tests based on this target had a clinical sensitivity of 63–98% and clinical specificity of 82.1–100%. [18, 33–36] This sequence has limitations despite the high sensitivity. For example, some strains of *M. tuberculosis* have been found which lack the *IS6110* sequence [17]. Furthermore, our in silico analysis with blastn showed that large fragments of this sequence have similarities with other NTM bacteria (*M. rutilum*, *M. smegmatis*, *M. chimaera*) and some Nocardia species such as *N. brasiliensis*. The 5KST sequence has only one copy in the genome of MTBC, nevertheless, it acts completely specific. In addition, unlike the *IS6110*, the 5KST is quite long which allows to design highly effective primers with minimal hairpin and dimer structures. Although the result of poorly designed primers may not appear in the test sensitivity of pure genomic DNA, it would quietly affect the clinical specimens and reduce the sensitivity [15].

Another diagnostic target sequence which many studies are based on, is *mpb64* [28, 37, 38]. This sequence has 687 bp length [39]. Our in silico study by blastn showed that it also contains large regions with high similarity to NTM species such as *M. kansasii*, *M. ulcerans*, and *M. hemophilum*. These nonspecific regions as well as the short length of sequence, make it very difficult to design efficient primers. As in most cases, the sensitivity reported for *mpb64* primers is lower than the *IS6110* primers [35]. An analytical sensitivity that eventually reported for 5KST-PCR was 1 copy per μl, which according to previous studies is better than *mpb64* -PCR (20 copies per μl) [18]. Also, *mpb64*-PCR had 88–91% specificity and 48–91% sensitivity on clinical specimens [18, 34, 35, 37].

*rpoB* diagnostic target has also been used to detect rifampicin-resistant TB in several studies [40]. Sensitivity and specificity of the PCR assays based on this target gene have been reported in two clinical trials about 93.3–95.8 and 100% respectively [41, 42]. However, our in silico analysis showed that this sequence has nonspecific similarities with some other mycobacteria. As the analytical specificity of this target in another study showed that the common primers designed for this sequence can also detect *M. chelonae*, *M. kansasii*, *M. scrofulaceum*, *M. smegmatis* and *M. szulgai* nonspecifically [18].

The *devR* sequence is another diagnostic target. In various studies, the detection limit of 200–500 copy per μl has been reported. This sequence has similarities with *M. kansasii* and may therefore results in false positives. In a clinical study of intraocular TB, same specificity and lower sensitivity compared to *mpb64* were reported [18, 43, 44].

In a comparative study between common diagnostic target sequences of TB, the sensitivity of *mpb64* was highest (84% in confirmed cases and 77.5% in clinically suspected cases). The clinical sensitivity of other targets was as follows: *mpb64* > *IS6110* > *hsp65* > 38KDa (*pstS1*) > 30KDa (*fbpB*) > *esat6* > *cfp10* > *devR* [5].

*sdaA* is another target sequence with few studies. This 1383 bp sequence encodes a protein called serine dehydratase. Although the detection limit equivalent to 1 copy per μl has been reported for this target [18], but our blastn results showed the presence of many nonspecific regions. This sequence has 70–80% similarity to some non-tuberculosis mycobacteria (*M. ulcerans*, *M. marinum*, *M. smegmatis*, and *M. fortuitum*) and many non-Mycobacterium bacteria such as Rhodococcus and Nocardia.

## Conclusions

In this study, we succeeded to introduce highly specific novel target using modified genome comparison method. We also designed highly specific primers, as they were able to detect even 1 copy of *M. tuberculosis* genomic DNA per μl. We think that the unique sensitivity of these primers is due to our long enough target which allows the software to give us the best possible primers.

Our recommendations for future studies include: 1. Using modified genome comparison method to identify diagnostic targets for other pathogens. 2. 5KST-PCR analysis on more and various clinical specimens. 3. Simultaneous comparison of 5KST-PCR with other PCR tests based on other targets to determine its efficiency. 4. Discovering the 5KST genes and their functions 5. Use of 5KST target in other diagnostic tests such as LAMP.

## Additional file

Additional file 1: **Table S1**. Investigating the presence of *rpoB* and 5KST diagnostic targets in *M. tuberculosis* complete genomes registered in nucleotide collection database (a part of NCBI). **Table S2**. Investigating the presence of 5KST sequence in strains collected from different parts of the world (TB Antibiotic Resistance Catalog project: https://olive.broadinstitute.org/projects/tb_arc/tree). **Figure S1**. Gel electrophoresis of 5KST-PCR products. It shows target amplicons at different concentrations of *M. tuberculosis* H37Rv genomic DNA spiked in processed clinical specimen as the template. (DOCX 250 kb)

## Abbreviations

LAMP: loop-mediated isothermal amplification;
MTBC: Mycobacterium tuberculosis complex
NCBI: National Center for Biotechnology Information
NTM: Nontuberculous Mycobacteria
PCR: Polymerase Chain Reaction
TB-ARC: Tuberculosis Antibiotic Resistance Catalog
