# Supplementary material for: Modified genome comparison method: a new approach for identification of specific targets in molecular diagnostic tests using Mycobacterium tuberculosis complex as an example
Source: BMC Infect Dis. 2018 Oct 12;18:517. doi: 10.1186/s12879-018-3417-x (PMC6186143; doi:10.1186/s12879-018-3417-x)
Supplement: Supplementary file 1 — Table S1. Investigating the presence of rpoB and 5KST diagnostic targets in M. tuberculosis complete genomes registered in nucleotide collection database (a part of NCBI). Table S2. Investigating the presence of 5KST sequence in strains collected from different parts of the world (TB Antibiotic Resistance Catalog project: https://olive.broadinstitute.org/projects/tb_arc/tree). Figure S1. Gel electrophoresis of 5KST-PCR products. It shows target amplicons at different concentrations of M. tuberculosis H37Rv genomic DNA spiked in processed clinical specimen as the template. (DOCX 250 kb) [file 12879_2018_3417_MOESM1_ESM.docx]

**Table S1.** Investigating the presence of rpoB and 5KST diagnostic targets in M. tuberculosis complete genomes registered in nucleotide collection database (a part of NCBI)

| **Name** | ***rpoB*** | **5KST** | **Name** | ***rpoB*** | **5KST** |
| --- | --- | --- | --- | --- | --- |
| 1. Mycobacterium tuberculosis 1821ADB35 genome |  |  | 1. Mycobacterium tuberculosis strain H112 chromosome, complete genome |  |  |
| 1. Mycobacterium tuberculosis 1821ADB36 genome |  |  | 1. Mycobacterium tuberculosis strain H54 chromosome, complete genome |  |  |
| 1. Mycobacterium tuberculosis 1821ADB37 genome |  |  | 1. Mycobacterium tuberculosis strain H83 chromosome, complete genome |  |  |
| 1. Mycobacterium tuberculosis 1821ADB38 genome |  |  | 1. Mycobacterium tuberculosis strain I0002353-6, complete genome |  |  |
| 1. Mycobacterium tuberculosis 1821ADB40 genome |  |  | 1. Mycobacterium tuberculosis strain I0002801-4, complete genome |  |  |
| 1. Mycobacterium tuberculosis 1821ADB41 genome |  |  | 1. Mycobacterium tuberculosis strain I0004000-1, complete genome |  |  |
| 1. Mycobacterium tuberculosis 1821ADB42 genome |  |  | 1. Mycobacterium tuberculosis strain I0004241-1, complete genome |  |  |
| 1. Mycobacterium tuberculosis 1821ADB44 genome |  |  | 1. Mycobacterium tuberculosis strain KIT87190, complete genome |  |  |
| 1. Mycobacterium tuberculosis 1821ADB45 genome |  |  | 1. Mycobacterium tuberculosis strain LE103 chromosome, complete genome |  |  |
| 1. Mycobacterium tuberculosis 18b genome |  |  | 1. Mycobacterium tuberculosis strain LE13 chromosome, complete genome |  |  |
| 1. Mycobacterium tuberculosis 49-02 complete genome |  |  | 1. Mycobacterium tuberculosis strain LE371 chromosome, complete genome |  |  |
| 1. Mycobacterium tuberculosis 7199-99 complete genome |  |  | 1. Mycobacterium tuberculosis strain LE410 chromosome, complete genome |  |  |
| 1. Mycobacterium tuberculosis BT1, complete genome |  |  | 1. Mycobacterium tuberculosis strain LE486 chromosome, complete genome |  |  |
| 1. Mycobacterium tuberculosis BT2, complete genome |  |  | 1. Mycobacterium tuberculosis strain LE492 chromosome, complete genome |  |  |
| 1. Mycobacterium tuberculosis CAS/NITR204, complete genome |  |  | 1. Mycobacterium tuberculosis strain LE63 chromosome, complete genome |  |  |
| 1. Mycobacterium tuberculosis CCDC5079, complete genome |  |  | 1. Mycobacterium tuberculosis strain LE76 chromosome, complete genome |  |  |
| 1. Mycobacterium tuberculosis CCDC5079, complete genome |  |  | 1. Mycobacterium tuberculosis strain LE79 chromosome, complete genome |  |  |
| 1. Mycobacterium tuberculosis CCDC5180, complete genome |  |  | 1. Mycobacterium tuberculosis strain LJ319 chromosome |  |  |
| 1. Mycobacterium tuberculosis CCDC5180, complete genome |  |  | 1. Mycobacterium tuberculosis strain LJ338 chromosome |  |  |
| 1. Mycobacterium tuberculosis CDC1551, complete genome |  |  | 1. Mycobacterium tuberculosis strain LN1100 chromosome, complete genome |  |  |
| 1. Mycobacterium tuberculosis CTRI-2, complete genome |  |  | 1. Mycobacterium tuberculosis strain LN180 chromosome, complete genome |  |  |
| 1. Mycobacterium tuberculosis DNA, complete genome, strain: HN-024 |  |  | 1. Mycobacterium tuberculosis strain LN1856 chromosome, complete genome |  |  |
| 1. Mycobacterium tuberculosis DNA, complete genome, strain: HN-205 |  |  | 1. Mycobacterium tuberculosis strain LN2358 chromosome, complete genome |  |  |
| 1. Mycobacterium tuberculosis DNA, complete genome, strain: HN-321 |  |  | 1. Mycobacterium tuberculosis strain LN2900 chromosome, complete genome |  |  |
| 1. Mycobacterium tuberculosis DNA, complete genome, strain: HN-506 |  |  | 1. Mycobacterium tuberculosis strain LN2978 chromosome, complete genome |  |  |
| 1. Mycobacterium tuberculosis DNA, complete genome, strain: NCGM946K2 |  |  | 1. Mycobacterium tuberculosis strain LN317 chromosome, complete genome |  |  |
| 1. Mycobacterium tuberculosis EAI5, complete genome |  |  | 1. Mycobacterium tuberculosis strain LN3584 chromosome, complete genome |  |  |
| 1. Mycobacterium tuberculosis EAI5/NITR206, complete genome |  |  | 1. Mycobacterium tuberculosis strain LN3588 chromosome, complete genome |  |  |
| 1. Mycobacterium tuberculosis F11, complete genome |  |  | 1. Mycobacterium tuberculosis strain LN3589 chromosome, complete genome |  |  |
| 1. Mycobacterium tuberculosis H37Ra, complete genome |  |  | 1. Mycobacterium tuberculosis strain LN3668 chromosome, complete genome |  |  |
| 1. Mycobacterium tuberculosis H37Ra, complete genome |  |  | 1. Mycobacterium tuberculosis strain LN3672 chromosome, complete genome |  |  |
| 1. Mycobacterium tuberculosis H37Rv complete genome |  |  | 1. Mycobacterium tuberculosis strain LN3695 chromosome, complete genome |  |  |
| 1. Mycobacterium tuberculosis H37Rv, complete genome |  |  | 1. Mycobacterium tuberculosis strain LN3756 chromosome, complete genome |  |  |
| 1. Mycobacterium tuberculosis H37Rv, complete genome |  |  | 1. Mycobacterium tuberculosis strain LN55 chromosome, complete genome |  |  |
| 1. Mycobacterium tuberculosis H37RvSiena, complete genome |  |  | 1. Mycobacterium tuberculosis strain LN763 chromosome, complete genome |  |  |
| 1. Mycobacterium tuberculosis HKBS1, complete genome |  |  | 1. Mycobacterium tuberculosis strain M0002959-6, complete genome |  |  |
| 1. Mycobacterium tuberculosis K, complete genome |  |  | 1. Mycobacterium tuberculosis strain M0018684-2, complete genome |  |  |
| 1. Mycobacterium tuberculosis KZN 1435, complete genome |  |  | 1. Mycobacterium tuberculosis strain MDRDM1098 chromosome, complete |  |  |
| 1. Mycobacterium tuberculosis KZN 4207, complete genome |  |  | 1. Mycobacterium tuberculosis strain MDRDM260 chromosome, complete |  |  |
| 1. Mycobacterium tuberculosis KZN 605, complete genome |  |  | 1. Mycobacterium tuberculosis strain MDRDM627 chromosome, complete |  |  |
| 1. Mycobacterium tuberculosis RGTB327, complete genome |  |  | 1. Mycobacterium tuberculosis strain MDRDM827 chromosome, complete |  |  |
| 1. Mycobacterium tuberculosis RGTB423, complete genome |  |  | 1. Mycobacterium tuberculosis strain MDRMA1565 chromosome, complete |  |  |
| 1. Mycobacterium tuberculosis str. Beijing/NITR203, complete genome |  |  | 1. Mycobacterium tuberculosis strain MDRMA2019 chromosome, complete |  |  |
| 1. Mycobacterium tuberculosis str. Erdman = ATCC 35801 DNA, |  |  | 1. Mycobacterium tuberculosis strain MDRMA203 chromosome, complete |  |  |
| 1. Mycobacterium tuberculosis str. Haarlem, complete genome |  |  | 1. Mycobacterium tuberculosis strain MDRMA2082 chromosome, complete |  |  |
| 1. Mycobacterium tuberculosis str. Haarlem/NITR202, complete genome |  |  | 1. Mycobacterium tuberculosis strain MDRMA2260 chromosome, complete |  |  |
| 1. Mycobacterium tuberculosis str. Kurono DNA, complete genome |  |  | 1. Mycobacterium tuberculosis strain MDRMA2441 chromosome, complete |  |  |
| 1. Mycobacterium tuberculosis strain 0A005DS genome |  |  | 1. Mycobacterium tuberculosis strain MDRMA2491 chromosome, complete |  |  |
| 1. Mycobacterium tuberculosis strain 0A029DS genome |  |  | 1. Mycobacterium tuberculosis strain MDRMA701 chromosome, complete |  |  |
| 1. Mycobacterium tuberculosis strain 0A033DS genome |  |  | 1. Mycobacterium tuberculosis strain MDRMA863 chromosome, complete |  |  |
| 1. Mycobacterium tuberculosis strain 0A036DS genome |  |  | 1. Mycobacterium tuberculosis strain ME1473 chromosome, complete genome |  |  |
| 1. Mycobacterium tuberculosis strain 0A087DS genome |  |  | 1. Mycobacterium tuberculosis strain MTB1, complete genome |  |  |
| 1. Mycobacterium tuberculosis strain 0A092DS genome |  |  | 1. Mycobacterium tuberculosis strain MTB2, complete genome |  |  |
| 1. Mycobacterium tuberculosis strain 0A093DS genome |  |  | 1. Mycobacterium tuberculosis strain MYC004 chromosome |  |  |
| 1. Mycobacterium tuberculosis strain 0A094DS genome |  |  | 1. Mycobacterium tuberculosis strain PR08 genome |  |  |
| 1. Mycobacterium tuberculosis strain 0A115DS genome |  |  | 1. Mycobacterium tuberculosis strain PR10 genome |  |  |
| 1. Mycobacterium tuberculosis strain 0A117DS genome |  |  | 1. Mycobacterium tuberculosis strain RUS_B0 chromosome, complete genome |  |  |
| 1. Mycobacterium tuberculosis strain 0B026XDR genome |  |  | 1. Mycobacterium tuberculosis strain S3 chromosome |  |  |
| 1. Mycobacterium tuberculosis strain 0B049XDR genome |  |  | 1. Mycobacterium tuberculosis strain SCAID 187.0, complete genome |  |  |
| 1. Mycobacterium tuberculosis strain 0B070XDR genome |  |  | 1. Mycobacterium tuberculosis strain SCAID 252.0, complete genome |  |  |
| 1. Mycobacterium tuberculosis strain 0B076XDR genome |  |  | 1. Mycobacterium tuberculosis strain SCAID 320.0, complete genome |  |  |
| 1. Mycobacterium tuberculosis strain 0B123ND genome |  |  | 1. Mycobacterium tuberculosis strain SLM036 chromosome, complete genome |  |  |
| 1. Mycobacterium tuberculosis strain 0B169XDR genome |  |  | 1. Mycobacterium tuberculosis strain SLM040 chromosome, complete genome |  |  |
| 1. Mycobacterium tuberculosis strain 0B218DS genome |  |  | 1. Mycobacterium tuberculosis strain SLM056 chromosome, complete genome |  |  |
| 1. Mycobacterium tuberculosis strain 0B222DS genome |  |  | 1. Mycobacterium tuberculosis strain SLM060 chromosome, complete genome |  |  |
| 1. Mycobacterium tuberculosis strain 0B228DS genome |  |  | 1. Mycobacterium tuberculosis strain SLM063 chromosome, complete genome |  |  |
| 1. Mycobacterium tuberculosis strain 0B229DS genome |  |  | 1. Mycobacterium tuberculosis strain SLM088 chromosome, complete genome |  |  |
| 1. Mycobacterium tuberculosis strain 0B235DS genome |  |  | 1. Mycobacterium tuberculosis strain SLM100 chromosome, complete genome |  |  |
| 1. Mycobacterium tuberculosis strain 0B259XDR genome |  |  | 1. Mycobacterium tuberculosis strain TB282, complete genome |  |  |
| 1. Mycobacterium tuberculosis strain 0B329XDR genome |  |  | 1. Mycobacterium tuberculosis strain TBDM1506 chromosome, complete genome |  |  |
| 1. Mycobacterium tuberculosis strain 1458, complete genome |  |  | 1. Mycobacterium tuberculosis strain TBDM2189 chromosome, complete genome |  |  |
| 1. Mycobacterium tuberculosis strain 22103, complete genome |  |  | 1. Mycobacterium tuberculosis strain TBDM2444 chromosome, complete genome |  |  |
| 1. Mycobacterium tuberculosis strain 22115, complete genome |  |  | 1. Mycobacterium tuberculosis strain TBDM2487 chromosome, complete genome |  |  |
| 1. Mycobacterium tuberculosis strain 2242, complete genome |  |  | 1. Mycobacterium tuberculosis strain TBDM2489 chromosome, complete genome |  |  |
| 1. Mycobacterium tuberculosis strain 2279, complete genome |  |  | 1. Mycobacterium tuberculosis strain TBDM2699 chromosome, complete genome |  |  |
| 1. Mycobacterium tuberculosis strain 26105, complete genome |  |  | 1. Mycobacterium tuberculosis strain TBDM2717 chromosome, complete genome |  |  |
| 1. Mycobacterium tuberculosis strain 37004, complete genome |  |  | 1. Mycobacterium tuberculosis strain TBDM425 chromosome, complete genome |  |  |
| 1. Mycobacterium tuberculosis strain 6A024XDR genome |  |  | 1. Mycobacterium tuberculosis strain TBMENG-03 chromosome, complete |  |  |
| 1. Mycobacterium tuberculosis strain 96075, complete genome |  |  | 1. Mycobacterium tuberculosis strain TBV4766 chromosome, complete genome |  |  |
| 1. Mycobacterium tuberculosis strain 96121, complete genome |  |  | 1. Mycobacterium tuberculosis strain TBV4768 chromosome, complete genome |  |  |
| 1. Mycobacterium tuberculosis strain Beijing, complete genome |  |  | 1. Mycobacterium tuberculosis strain TBV4952 chromosome, complete genome |  |  |
| 1. Mycobacterium tuberculosis strain Beijing/391, complete genome |  |  | 1. Mycobacterium tuberculosis strain TBV5000 chromosome, complete genome |  |  |
| 1. Mycobacterium tuberculosis strain Beijing2014PNGD chromosome |  |  | 1. Mycobacterium tuberculosis strain TBV5362 chromosome, complete genome |  |  |
| 1. Mycobacterium tuberculosis strain Beijing-like, complete genome |  |  | 1. Mycobacterium tuberculosis strain TBV5365 chromosome, complete genome |  |  |
| 1. Mycobacterium tuberculosis strain Beijing-like/1104, complete genome |  |  | 1. Mycobacterium tuberculosis strain WC059 chromosome, complete genome |  |  |
| 1. Mycobacterium tuberculosis strain Beijing-like/35049, complete genome |  |  | 1. Mycobacterium tuberculosis strain WC078 chromosome, complete genome |  |  |
| 1. Mycobacterium tuberculosis strain Beijing-like/36918, complete genome |  |  | 1. Mycobacterium tuberculosis strain ZMC13-264, complete genome |  |  |
| 1. Mycobacterium tuberculosis strain Beijing-like/38774, complete genome |  |  | 1. Mycobacterium tuberculosis strain ZMC13-88, complete genome |  |  |
| 1. Mycobacterium tuberculosis strain Beijing-like/50148, complete genome |  |  | 1. Mycobacterium tuberculosis TRS1 genome |  |  |
| 1. Mycobacterium tuberculosis strain C3 chromosome |  |  | 1. Mycobacterium tuberculosis TRS10 genome |  |  |
| 1. Mycobacterium tuberculosis strain CAS chromosome |  |  | 1. Mycobacterium tuberculosis TRS11 genome |  |  |
| 1. Mycobacterium tuberculosis strain CSV10399 chromosome, complete |  |  | 1. Mycobacterium tuberculosis TRS12 genome |  |  |
| 1. Mycobacterium tuberculosis strain CSV11678 chromosome |  |  | 1. Mycobacterium tuberculosis TRS13 genome |  |  |
| 1. Mycobacterium tuberculosis strain CSV3611 chromosome, complete |  |  | 1. Mycobacterium tuberculosis TRS14 genome |  |  |
| 1. Mycobacterium tuberculosis strain CSV383 chromosome, complete |  |  | 1. Mycobacterium tuberculosis TRS15 genome |  |  |
| 1. Mycobacterium tuberculosis strain CSV4519 chromosome, complete |  |  | 1. Mycobacterium tuberculosis TRS16 genome |  |  |
| 1. Mycobacterium tuberculosis strain CSV4644 chromosome, complete |  |  | 1. Mycobacterium tuberculosis TRS17 genome |  |  |
| 1. Mycobacterium tuberculosis strain CSV5769 chromosome, complete |  |  | 1. Mycobacterium tuberculosis TRS18 genome |  |  |
| 1. Mycobacterium tuberculosis strain CSV9577 chromosome, complete |  |  | 1. Mycobacterium tuberculosis TRS19 genome |  |  |
| 1. Mycobacterium tuberculosis strain DK9897, complete genome |  |  | 1. Mycobacterium tuberculosis TRS2 genome |  |  |
| 1. Mycobacterium tuberculosis strain F1, complete genome |  |  | 1. Mycobacterium tuberculosis TRS20 genome |  |  |
| 1. Mycobacterium tuberculosis strain F28, complete genome |  |  | 1. Mycobacterium tuberculosis TRS21 genome |  |  |
| 1. Mycobacterium tuberculosis strain GG-109-10 chromosome, complete |  |  | 1. Mycobacterium tuberculosis TRS22 genome |  |  |
| 1. Mycobacterium tuberculosis strain GG-111-10 chromosome, complete |  |  | 1. Mycobacterium tuberculosis TRS23 genome |  |  |
| 1. Mycobacterium tuberculosis strain GG-121-10 chromosome, complete |  |  | 1. Mycobacterium tuberculosis TRS24 genome |  |  |
| 1. Mycobacterium tuberculosis strain GG-129-11 chromosome, complete |  |  | 1. Mycobacterium tuberculosis TRS25 genome |  |  |
| 1. Mycobacterium tuberculosis strain GG-134-11 chromosome, complete |  |  | 1. Mycobacterium tuberculosis TRS26 genome |  |  |
| 1. Mycobacterium tuberculosis strain GG-137-10 chromosome, complete |  |  | 1. Mycobacterium tuberculosis TRS27 genome |  |  |
| 1. Mycobacterium tuberculosis strain GG-186-10 chromosome, complete |  |  | 1. Mycobacterium tuberculosis TRS28 genome |  |  |
| 1. Mycobacterium tuberculosis strain GG-20-11 chromosome, complete |  |  | 1. Mycobacterium tuberculosis TRS29 genome |  |  |
| 1. Mycobacterium tuberculosis strain GG-229-10 chromosome, complete |  |  | 1. Mycobacterium tuberculosis TRS4 genome |  |  |
| 1. Mycobacterium tuberculosis strain GG-27-11 chromosome, complete |  |  | 1. Mycobacterium tuberculosis TRS5 genome |  |  |
| 1. Mycobacterium tuberculosis strain GG-36-11 chromosome, complete |  |  | 1. Mycobacterium tuberculosis TRS6 genome |  |  |
| 1. Mycobacterium tuberculosis strain GG-37-11 chromosome, complete |  |  | 1. Mycobacterium tuberculosis TRS7 genome |  |  |
| 1. Mycobacterium tuberculosis strain GG-45-11 chromosome, complete |  |  | 1. Mycobacterium tuberculosis TRS8 genome |  |  |
| 1. Mycobacterium tuberculosis strain GG-5-10 chromosome, complete |  |  | 1. Mycobacterium tuberculosis TRS9 genome |  |  |
| 1. Mycobacterium tuberculosis strain GG-77-11 chromosome, complete |  |  | 1. Mycobacterium tuberculosis UT205 complete genome |  |  |
| 1. Mycobacterium tuberculosis strain GG-90-10 chromosome, complete |  |  | 1. Mycobacterium tuberculosis W-148, complete genome |  |  |
| 1. Mycobacterium tuberculosis strain H107 chromosome, complete genome |  |  |  |  |  |

**Table S2.** Investigating the presence of 5KST sequence in strains collected from different parts of the world (TB Antibiotic Resistance Catalog project: <https://olive.broadinstitute.org/projects/tb_arc/tree>)

| India  "TB-ARC India initiative, Broad Institute (broadinstitute.org)" | | | | Sweden  "TB-ARC Sweden initiative, Broad Institute (broadinstitute.org)" | | | |
| --- | --- | --- | --- | --- | --- | --- | --- |
|  | name | Query cover | ident |  | name | Query cover | ident |
| 1 | mycobacterium_tuberculosis_h1249.0.scaffolds | 100 | 99 | 1 | mycobacterium_tuberculosis_btb03_012.0.scaffolds | 100 | 99 |
| 2 | mycobacterium_tuberculosis_h1580.0.scaffolds | 100 | 99 | 2 | mycobacterium_tuberculosis_btb03_143.0.scaffolds | 85 | 99 |
| 3 | mycobacterium_tuberculosis_h1996.0.scaffolds | 100 | 99 | 3 | mycobacterium_tuberculosis_btb03_169.0.scaffolds | 100 | 99 |
| 4 | mycobacterium_tuberculosis_h2264.0.scaffolds | 100 | 99 | 4 | mycobacterium_tuberculosis_btb04_120.0.scaffolds | 100 | 100 |
| 5 | mycobacterium_tuberculosis_h2463.0.scaffolds | 100 | 99 | 5 | mycobacterium_tuberculosis_btb04_128.0.scaffolds | 100 | 99 |
| 6 | mycobacterium_tuberculosis_h2760.0.scaffolds | 100 | 99 | 6 | mycobacterium_tuberculosis_btb04_388.0.scaffolds | 100 | 99 |
| 7 | mycobacterium_tuberculosis_m13.0.scaffolds | 100 | 99 | 7 | mycobacterium_tuberculosis_btb05_001.0.scaffolds | 100 | 99 |
| 8 | mycobacterium_tuberculosis_m984.0.scaffolds | 100 | 99 | 8 | mycobacterium_tuberculosis_btb05_481.0.scaffolds | 100 | 100 |
| 9 | mycobacterium_tuberculosis_m1007.0.scaffolds | 100 | 99 | 9 | mycobacterium_tuberculosis_btb05_660.0.scaffolds | 100 | 99 |
| 10 | mycobacterium_tuberculosis_m1010.0.scaffolds | 100 | 99 | 10 | mycobacterium_tuberculosis_btb06_467.0.scaffolds | 100 | 99 |
| 11 | mycobacterium_tuberculosis_m1017.0.scaffolds | 100 | 99 | 11 | mycobacterium_tuberculosis_btb07_001.0.scaffolds | 100 | 99 |
| 12 | mycobacterium_tuberculosis_m1022.0.scaffolds | 100 | 99 | 12 | mycobacterium_tuberculosis_btb07_170.0.scaffolds | 85 | 99 |
| 13 | mycobacterium_tuberculosis_m1025.0.scaffolds | 100 | 99 | 13 | mycobacterium_tuberculosis_btb07_327.0.scaffolds | 100 | 99 |
| 14 | mycobacterium_tuberculosis_m1210.0.scaffolds | 100 | 99 | 14 | mycobacterium_tuberculosis_btb07_354.0.scaffolds | 100 | 99 |
| 15 | mycobacterium_tuberculosis_m1213.0.scaffolds | 100 | 99 | 15 | mycobacterium_tuberculosis_btb08_043.0.scaffolds | 100 | 99 |
| 16 | mycobacterium_tuberculosis_m1236.0.scaffolds | 100 | 99 | 16 | mycobacterium_tuberculosis_btb08_183.0.scaffolds | 85 | 99 |
| 17 | mycobacterium_tuberculosis_m1272.0.scaffolds | 100 | 99 | 17 | mycobacterium_tuberculosis_btb08_221.0.scaffolds | 100 | 99 |
| 18 | mycobacterium_tuberculosis_m1288.0.scaffolds | 100 | 99 | 18 | mycobacterium_tuberculosis_btb08_356.0.scaffolds | 100 | 99 |
| 19 | mycobacterium_tuberculosis_m1294.0.scaffolds | 100 | 99 | 19 | mycobacterium_tuberculosis_btb09_036.0.scaffolds | 100 | 99 |
| 20 | mycobacterium_tuberculosis_m1309.0.scaffolds | 100 | 99 | 20 | mycobacterium_tuberculosis_btb10_001.0.scaffolds | 100 | 99 |
| 21 | mycobacterium_tuberculosis_m1317.0.scaffolds | 100 | 99 | 21 | mycobacterium_tuberculosis_btb10_092.0.scaffolds | 100 | 99 |
| 22 | mycobacterium_tuberculosis_m1324.0.scaffolds | 100 | 99 | 22 | mycobacterium_tuberculosis_btb10_120.0.scaffolds | 100 | 99 |
| 23 | mycobacterium_tuberculosis_m1373.0.scaffolds | 100 | 99 | 22 | mycobacterium_tuberculosis_btb10_142.0.scaffolds | 100 | 99 |
| 24 | mycobacterium_tuberculosis_m1392.0.scaffolds | 100 | 99 | 23 | mycobacterium_tuberculosis_btb10_277.0.scaffolds | 100 | 99 |
| 25 | mycobacterium_tuberculosis_m1400.0.scaffolds | 100 | 99 | 24 | mycobacterium_tuberculosis_btb10_422.0.scaffolds | 100 | 99 |
| 26 | mycobacterium_tuberculosis_m1410.0.scaffolds | 100 | 99 | 25 | mycobacterium_tuberculosis_btb11_001.0.scaffolds | 100 | 99 |
| 27 | mycobacterium_tuberculosis_m1417.0.scaffolds | 100 | 99 | 26 | mycobacterium_tuberculosis_btb11_207.0.scaffolds | 100 | 99 |
| 28 | mycobacterium_tuberculosis_m1425.0.scaffolds | 100 | 99 | 27 | mycobacterium_tuberculosis_btb11_210.0.scaffolds | 100 | 99 |
| 29 | mycobacterium_tuberculosis_m1438.0.scaffolds | 100 | 99 | 28 | mycobacterium_tuberculosis_btb11_371.0.scaffolds | 83 | 99 |
| 30 | mycobacterium_tuberculosis_m1441.0.scaffolds | 100 | 99 | 29 | mycobacterium_tuberculosis_btb12_046.0.scaffolds | 100 | 99 |
| 31 | mycobacterium_tuberculosis_m1456.0.scaffolds | 100 | 99 | 30 | mycobacterium_tuberculosis_btb12_162.0.scaffolds | 100 | 99 |
| 32 | mycobacterium_tuberculosis_m1475.0.scaffolds | 100 | 99 | 31 | mycobacterium_tuberculosis_btb12_211.0.scaffolds | 83 | 99 |
| 33 | mycobacterium_tuberculosis_m1533.0.scaffolds | 100 | 99 | 32 | mycobacterium_tuberculosis_btb12_294.0.scaffolds | 100 | 99 |
| 34 | mycobacterium_tuberculosis_m1570.0.scaffolds | 100 | 99 | 33 | mycobacterium_tuberculosis_btb12_313.0.scaffolds | 100 | 99 |
| 35 | mycobacterium_tuberculosis_m1702.0.scaffolds | 100 | 99 | 34 | mycobacterium_tuberculosis_btb12_314.0.scaffolds | 85 | 99 |
| 36 | mycobacterium_tuberculosis_m1734.0.scaffolds | 100 | 99 | 35 | mycobacterium_tuberculosis_btb13_001.0.scaffolds | 100 | 99 |
| 37 | mycobacterium_tuberculosis_m1782.0.scaffolds | 81 | 99 | 36 | mycobacterium_tuberculosis_btb13_156.0.scaffolds |  |  |
| 38 | mycobacterium_tuberculosis_m1848.0.scaffolds | 81 | 99 | Iran  "TB-ARC Iran initiative, Broad Institute (broadinstitute.org)" | |  |  |
| 39 | mycobacterium_tuberculosis_m1906.0.scaffolds | 100 | 99 |  |  |  |  |
| 40 | mycobacterium_tuberculosis_m1926.0.scaffolds | 81 | 99 |  |  |  |  |
| 41 | mycobacterium_tuberculosis_m1932.0.scaffolds | 81 | 99 |  |  |  |  |
| 42 | mycobacterium_tuberculosis_m1956.0.scaffolds | 81 | 99 | name | | Query cover | ident |
| 43 | mycobacterium_tuberculosis_m1967.0.scaffolds | 100 | 99 | 1 | mycobacterium_tuberculosis_nritld33.0.scaffolds | 100 | 99 |
| 44 | mycobacterium_tuberculosis_m1979.0.scaffolds | 100 | 99 | 2 | mycobacterium_tuberculosis_nritld34.0.scaffolds | 100 | 100 |
| 45 | mycobacterium_tuberculosis_m2007.0.scaffolds | 100 | 99 | 3 | mycobacterium_tuberculosis_nritld37.0.scaffolds | 82 | 99 |
| 46 | mycobacterium_tuberculosis_m2021.0.scaffolds | 100 | 99 | 4 | mycobacterium_tuberculosis_nritld38.0.scaffolds | 83 | 99 |
| 47 | mycobacterium_tuberculosis_m2085.0.scaffolds | 81 | 99 | 5 | mycobacterium_tuberculosis_nritld45.0.scaffolds | 100 | 99 |
| 48 | mycobacterium_tuberculosis_m2116.0.scaffolds | 100 | 99 | 6 | mycobacterium_tuberculosis_nritld59.0.scaffolds | 82 | 99 |
| 49 | mycobacterium_tuberculosis_m2137.0.scaffolds | 100 | 99 | 7 | mycobacterium_tuberculosis_nritld60.0.scaffolds | 100 | 100 |
| 50 | mycobacterium_tuberculosis_m2144.0.scaffolds | 100 | 99 | 8 | mycobacterium_tuberculosis_nritld28.0.scaffolds | 100 | 99 |
| 51 | mycobacterium_tuberculosis_m2211.0.scaffolds | 100 | 99 |  | | | |
| 52 | mycobacterium_tuberculosis_m2278.0.scaffolds | 100 | 99 |  |  |  |  |
| 53 | mycobacterium_tuberculosis_m2343.0.scaffolds | 100 | 99 |  |  |  |  |
|  | | | |  |  |  |  |
| Mali  "TB-ARC Mali initiative, Broad Institute (broadinstitute.org)" | | | | USA (Mycobacterium bovis group)  "TB-ARC M. bovis initiative, Broad Institute (broadinstitute.org)" | | | |
|  | name | Query cover | ident |  | name | Query cover | ident |
| 1 | mycobacterium_africanum_mal010070.0.scaffolds | 100 | 99 | 1 | mycobacterium_bovis_b2_7505.0.scaffolds | 100 | 99 |
| 2 | mycobacterium_africanum_mal010071.0.scaffolds | 100 | 99 | 2 | mycobacterium_bovis_bz_31150.0.scaffolds | 100 | 99 |
| 3 | mycobacterium_africanum_mal010074.0.scaffolds | 100 | 99 | 3 | mycobacterium_bovis_d_4155.0.scaffolds | 100 | 99 |
| 4 | mycobacterium_africanum_mal010079.0.scaffolds | 100 | 99 | 4 | mycobacterium_bovis_kc_9614.0.scaffolds | 100 | 99 |
| 5 | mycobacterium_africanum_mal010081.0.scaffolds | 100 | 99 | 5 | mycobacterium_bovis_kc_32216.0.scaffolds | 100 | 99 |
| 6 | mycobacterium_africanum_mal010084.0.scaffolds | 100 | 99 | 6 | mycobacterium_bovis_mr_4387.0.scaffolds | 100 | 99 |
| 7 | mycobacterium_africanum_mal010099.0.scaffolds | 100 | 99 | 7 | mycobacterium_bovis_wt_21231.0.scaffolds | 100 | 100 |
| 8 | mycobacterium_africanum_mal010100.0.scaffolds | 100 | 99 | 8 | mycobacterium_bovis_wt_21419.0.scaffolds | 100 | 99 |
| 9 | mycobacterium_africanum_mal010102.0.scaffolds | 100 | 99 |  |  |  |  |
| 10 | mycobacterium_africanum_mal010111.0.scaffolds | 100 | 99 |  |  |  |  |
| 11 | mycobacterium_africanum_mal010112.0.scaffolds | 100 | 99 |  |  |  |  |
| 12 | mycobacterium_africanum_mal010118.0.scaffolds | 100 | 99 | Uganda and South Korea  "TB-ARC CDRC Alland initiative, Broad Institute (broadinstitute.org)" | | | |
|  |  |  |  |  | name | Query cover | ident |
| 13 | mycobacterium_africanum_mal010120.0.scaffolds | 100 | 99 | 1 | mycobacterium_tuberculosis_kt_0002.0.scaffolds | 83 | 99 |
| 14 | mycobacterium_africanum_mal010123.0.scaffolds | 100 | 99 | 2 | mycobacterium_tuberculosis_kt_0003.0.scaffolds | 84 | 99 |
| 15 | mycobacterium_africanum_mal010128.0.scaffolds | 100 | 99 | 3 | mycobacterium_tuberculosis_kt_0024.0.scaffolds | 83 | 99 |
| 16 | mycobacterium_africanum_mal010129.0.scaffolds | 100 | 99 | 4 | mycobacterium_tuberculosis_kt_0033.0.scaffolds | 84 | 99 |
| 17 | mycobacterium_africanum_mal010131.0.scaffolds | 100 | 99 | 5 | mycobacterium_tuberculosis_kt_0039.0.scaffolds | 85 | 99 |
| 18 | mycobacterium_africanum_mal010136.0.scaffolds | 100 | 99 | 6 | mycobacterium_tuberculosis_kt_0045.0.scaffolds | 100 | 99 |
| 19 | mycobacterium_africanum_mal010137.0.scaffolds | 100 | 99 | 7 | mycobacterium_tuberculosis_kt_0048.0.scaffolds | 100 | 99 |
| 20 | mycobacterium_africanum_mal020107.0.scaffolds | 100 | 99 | 8 | mycobacterium_tuberculosis_kt_0051.0.scaffolds | 83 | 99 |
| 21 | mycobacterium_africanum_mal020130.0.scaffolds | 100 | 99 | 9 | mycobacterium_tuberculosis_kt_0056.0.scaffolds | 100 | 99 |
| 22 | mycobacterium_africanum_mal020135.0.scaffolds | 100 | 99 | 10 | mycobacterium_tuberculosis_kt_0057.0.scaffolds | 83 | 99 |
| 23 | mycobacterium_africanum_mal020148.0.scaffolds | 100 | 99 | 11 | mycobacterium_tuberculosis_kt_0064.0.scaffolds | 82 | 99 |
| 24 | mycobacterium_africanum_mal020173.0.scaffolds | 100 | 99 | 12 | mycobacterium_tuberculosis_kt_0071.0.scaffolds | 83 | 99 |
| 25 | mycobacterium_africanum_mal020176.0.scaffolds | 100 | 99 | 13 | mycobacterium_tuberculosis_kt_0075.0.scaffolds | 83 | 99 |
| 26 | mycobacterium_africanum_mal020185.0.scaffolds | 100 | 99 | 14 | mycobacterium_tuberculosis_kt_0079.0.scaffolds | 100 | 99 |
| 27 | mycobacterium_bovis_mal010093.0.scaffolds | 100 | 99 | 15 | mycobacterium_tuberculosis_kt_0083.0.scaffolds | 100 | 99 |
| Africa  "TB-ARC MRC SA initiative, Broad Institute (broadinstitute.org)" | | | | 16 | mycobacterium_tuberculosis_kt_0094.0.scaffolds | 83 | 99 |
|  | name | Query cover | ident | 17 | mycobacterium_tuberculosis_kt_0100.0.scaffolds | 83 | 99 |
| 1 | mycobacterium_tuberculosis_tb_rsa01.0.scaffolds | 83 | 99 | 18 | mycobacterium_tuberculosis_kt_0104.0.scaffolds | 83 | 99 |
| 2 | mycobacterium_tuberculosis_tb_rsa02.0.scaffolds | 83 | 99 | 19 | mycobacterium_tuberculosis_ug_d.0.scaffolds | 100 | 99 |
| 3 | mycobacterium_tuberculosis_tb_rsa03.0.scaffolds | 83 | 99 | 20 | mycobacterium_tuberculosis_ut0002.0.scaffolds | 100 | 99 |
| 4 | mycobacterium_tuberculosis_tb_rsa04.0.scaffolds | 85 | 99 | 21 | mycobacterium_tuberculosis_ut0009.0.scaffolds | 100 | 99 |
| 5 | mycobacterium_tuberculosis_tb_rsa05.0.scaffolds | 84 | 99 | 22 | mycobacterium_tuberculosis_ut0022.0.scaffolds | 100 | 99 |
| 6 | mycobacterium_tuberculosis_tb_rsa06.0.scaffolds | 84 | 99 | 23 | mycobacterium_tuberculosis_ut0026.0.scaffolds | 100 | 99 |
| 7 | mycobacterium_tuberculosis_tb_rsa07.0.scaffolds | 100 | 99 | 24 | mycobacterium_tuberculosis_ut0028.0.scaffolds | 100 | 99 |
| 8 | mycobacterium_tuberculosis_tb_rsa08.0.scaffolds | 100 | 99 | 25 | mycobacterium_tuberculosis_ut0037.0.scaffolds | 100 | 99 |
| 9 | mycobacterium_tuberculosis_tb_rsa09.0.scaffolds | 83 | 99 | 26 | mycobacterium_tuberculosis_ut0040.0.scaffolds | 100 | 99 |
| 10 | mycobacterium_tuberculosis_tb_rsa10.0.scaffolds | 85 | 99 | 27 | mycobacterium_tuberculosis_ut0044.0.scaffolds | 100 | 99 |
| 11 | mycobacterium_tuberculosis_tb_rsa11.0.scaffolds | 82 | 99 | 28 | mycobacterium_tuberculosis_ut0050.0.scaffolds | 85 | 99 |
| 12 | mycobacterium_tuberculosis_tb_rsa12.0.scaffolds | 82 | 99 | 29 | mycobacterium_tuberculosis_ut0051.0.scaffolds | 100 | 99 |
| 13 | mycobacterium_tuberculosis_tb_rsa13.0.scaffolds | 83 | 99 | 30 | mycobacterium_tuberculosis_ut0053.0.scaffolds | 100 | 99 |
| 14 | mycobacterium_tuberculosis_tb_rsa14.0.scaffolds | 85 | 99 | 31 | mycobacterium_tuberculosis_ut0054.0.scaffolds | 100 | 99 |
| 15 | mycobacterium_tuberculosis_tb_rsa15.0.scaffolds | 83 | 99 | 32 | mycobacterium_tuberculosis_ut0058.0.scaffolds | 100 | 99 |
| 16 | mycobacterium_tuberculosis_tb_rsa16.0.scaffolds | 83 | 99 | 33 | mycobacterium_tuberculosis_ut0069.0.scaffolds | 100 | 99 |
| 17 | mycobacterium_tuberculosis_tb_rsa17.0.scaffolds | 81 | 99 | 34 | mycobacterium_tuberculosis_ut0091.0.scaffolds | 82 | 99 |
| 18 | mycobacterium_tuberculosis_tb_rsa18.0.scaffolds | 83 | 99 | 35 | mycobacterium_tuberculosis_ut0093.0.scaffolds | 100 | 99 |
| 19 | mycobacterium_tuberculosis_tb_rsa19.0.scaffolds | 85 | 99 | 36 | mycobacterium_tuberculosis_ut0097.0.scaffolds | 100 | 99 |
| 20 | mycobacterium_tuberculosis_tb_rsa20.0.scaffolds | 83 | 99 | 37 | mycobacterium_tuberculosis_ut0106.0.scaffolds | 100 | 99 |
| 21 | mycobacterium_tuberculosis_tb_rsa21.0.scaffolds | 83 | 99 | 38 | mycobacterium_tuberculosis_ut0110.0.scaffolds | 100 | 99 |
| 22 | mycobacterium_tuberculosis_tb_rsa22.0.scaffolds | 81 | 99 | 39 | mycobacterium_tuberculosis_ut0115.0.scaffolds | 100 | 99 |
| 23 | mycobacterium_tuberculosis_tb_rsa23.0.scaffolds | 83 | 99 | 40 | mycobacterium_tuberculosis_ut0124.0.scaffolds | 100 | 99 |
| 24 | mycobacterium_tuberculosis_tb_rsa24.0.scaffolds | 82 | 99 | South Africa(KwaZulu-Natal)  "TB-ARC K-RITH initiative, Broad Institute (broadinstitute.org)" | | | |
| 25 | mycobacterium_tuberculosis_tb_rsa25.0.scaffolds | 100 | 99 |  | name | Query cover | ident |
| 26 | mycobacterium_tuberculosis_tb_rsa26.0.scaffolds | 100 | 99 | 1 | myco_sp_tkk-01-0051.1.scaffolds | 35 | 86 |
| 27 | mycobacterium_tuberculosis_tb_rsa27.0.scaffolds | 85 | 99 | 2 | myco_sp_tkk-01-0059.1.scaffolds | 30 | 78 |
| 28 | mycobacterium_tuberculosis_tb_rsa28.0.scaffolds | 81 | 99 | 3 | myco_tube_tkk_02_0001.i1.scaffolds | 85 | 99 |
| 29 | mycobacterium_tuberculosis_tb_rsa35.0.scaffolds | 83 | 99 | 4 | myco_tube_tkk_02_0025.i1.scaffolds | 100 | 99 |
| 30 | mycobacterium_tuberculosis_tb_rsa37.0.scaffolds | 83 | 99 | 5 | myco_tube_tkk_02_0034.i1.scaffolds | 100 | 99 |
| 31 | mycobacterium_tuberculosis_tb_rsa57.0.scaffolds | 100 | 99 | 6 | myco_tube_tkk_02_0062.i1.scaffolds | 100 | 99 |
| 32 | mycobacterium_tuberculosis_tb_rsa58.0.scaffolds | 100 | 99 | 7 | myco_tube_tkk_02_0063.i1.scaffolds | 83 | 99 |
| 33 | mycobacterium_tuberculosis_tb_rsa75.0.scaffolds | 100 | 99 | 8 | myco_tube_tkk_02_0066.1.scaffolds | 100 | 99 |
| 34 | mycobacterium_tuberculosis_tb_rsa76.0.scaffolds | 100 | 99 | 9 | myco_tube_tkk_02_0067.i1.scaffolds | 100 | 99 |
| 35 | mycobacterium_tuberculosis_tb_rsa89.0.scaffolds | 100 | 100 | 10 | myco_tube_tkk_02_0069.i1.scaffolds | 100 | 100 |
| 36 | mycobacterium_tuberculosis_tb_rsa90.0.scaffolds | 100 | 99 | 11 | myco_tube_tkk_02_0073.i1.scaffolds | 100 | 99 |
| 37 | mycobacterium_tuberculosis_tb_rsa95.0.scaffolds | 100 | 100 | 12 | myco_tube_tkk_03_0018.i1.scaffolds | 100 | 99 |
| 38 | mycobacterium_tuberculosis_tb_rsa98.0.scaffolds | 100 | 99 | 13 | myco_tube_tkk_03_0025.i1.scaffolds | 85 | 99 |
| 39 | mycobacterium_tuberculosis_tb_rsa99.0.scaffolds | 100 | 99 | 14 | myco_tube_tkk_03_0026.i1.scaffolds | 100 | 99 |
| 40 | mycobacterium_tuberculosis_tb_rsa107.0.scaffolds | 100 | 99 | 15 | myco_tube_tkk_03_0029.i1.scaffolds | 100 | 99 |
| 41 | mycobacterium_tuberculosis_tb_rsa113.0.scaffolds | 100 | 100 | 16 | myco_tube_tkk_03_0030.i1.scaffolds | 100 | 99 |
| 42 | mycobacterium_tuberculosis_tb_rsa149.0.scaffolds | 100 | 99 | 17 | myco_tube_tkk_03_0031.i1.scaffolds | 85 | 99 |
| 43 | mycobacterium_tuberculosis_tb_rsa169.0.scaffolds | 100 | 99 | 18 | myco_tube_tkk_03_0033.i1.scaffolds | 100 | 99 |
| 44 | mycobacterium_tuberculosis_tb_rsa195.0.scaffolds | 100 | 99 | 19 | myco_tube_tkk_03_0036.i1.scaffolds | 100 | 99 |
| 45 | mycobacterium_tuberculosis_tb_rsa101.0.scaffolds | 100 | 99 | 20 | myco_tube_tkk_04_0003.i1.scaffolds | 100 | 99 |
| 46 | mycobacterium_tuberculosis_tb_rsa103.0.scaffolds | 100 | 99 | 21 | myco_tube_tkk_04_0005.i1.scaffolds | 100 | 99 |
| 47 | mycobacterium_tuberculosis_tb_rsa123.0.scaffolds | 100 | 99 | 22 | myco_tube_tkk_04_0014.i1.scaffolds | 85 | 99 |
| 48 | mycobacterium_tuberculosis_tb_rsa131.0.scaffolds | 100 | 99 | 23 | myco_tube_tkk_04_0020.i1.scaffolds | 100 | 99 |
| 49 | mycobacterium_tuberculosis_tb_rsa134.0.scaffolds | 100 | 99 | 24 | myco_tube_tkk_04_0029.i1.scaffolds | 100 | 99 |
| 50 | mycobacterium_tuberculosis_tb_rsa168.0.scaffolds | 100 | 99 | 25 | myco_tube_tkk_04_0031.i1.scaffolds | 100 | 100 |
| 51 | mycobacterium_tuberculosis_tb_rsa189.0.scaffolds | 83 | 99 | 26 | myco_tube_tkk_04_0036.i1.scaffolds | 100 | 99 |
| Moldova  "TB-ARC Moldova initiative, Broad Institute (broadinstitute.org)" | | | | 27 | myco_tube_tkk_04_0047.i1.scaffolds | 100 | 99 |
|  | name | Query cover | ident | 28 | myco_tube_tkk-01-0007.1.scaffolds | 100 | 99 |
| 1 | mycobacterium_tuberculosis_md15766.0.scaffolds | 100 | 99 | 29 | myco_tube_tkk-01-0008.1.scaffolds | 85 | 99 |
| 2 | mycobacterium_tuberculosis_md15974.0.scaffolds | 100 | 99 | 30 | myco_tube_tkk-01-0009.1.scaffolds | 100 | 99 |
| 3 | mycobacterium_tuberculosis_md16775.0.scaffolds | 100 | 99 | 31 | myco_tube_tkk-01-0010.1.scaffolds | 100 | 99 |
| 4 | mycobacterium_tuberculosis_md17613.0.scaffolds | 100 | 99 | 32 | myco_tube_tkk-01-0018.1.scaffolds | 100 | 99 |
| 5 | mycobacterium_tuberculosis_md17615.0.scaffolds | 100 | 99 | 33 | myco_tube_tkk-01-0022.1.scaffolds | 100 | 99 |
| 6 | mycobacterium_tuberculosis_md17656.0.scaffolds | 100 | 99 | 34 | myco_tube_tkk-01-0027.1.scaffolds | 83 | 99 |
| 7 | mycobacterium_tuberculosis_md17902.0.scaffolds | 100 | 99 | 35 | myco_tube_tkk-01-0037.1.scaffolds | 100 | 99 |
| 8 | mycobacterium_tuberculosis_md17903.0.scaffolds | 100 | 99 | 36 | myco_tube_tkk-01-0039.1.scaffolds | 85 | 99 |
| 9 | mycobacterium_tuberculosis_md19693.0.scaffolds | 100 | 99 | 37 | myco_tube_tkk-01-0042.1.scaffolds | 100 | 99 |
| 10 | mycobacterium_tuberculosis_md15226.0.scaffolds | 100 | 99 | 38 | myco_tube_tkk-01-0048.1.scaffolds | 100 | 99 |
| 11 | mycobacterium_tuberculosis_md16265.0.scaffolds | 100 | 99 | 39 | myco_tube_tkk-01-0049.1.scaffolds | 100 | 99 |
| 12 | mycobacterium_tuberculosis_md16277.0.scaffolds | 100 | 99 | 40 | myco_tube_tkk-01-0050.1.scaffolds | 100 | 99 |
| 13 | mycobacterium_tuberculosis_md16728.0.scaffolds | 100 | 99 | 41 | myco_tube_tkk-01-0010.1.scaffolds | 100 | 99 |
| 14 | mycobacterium_tuberculosis_md17646.0.scaffolds | 100 | 99 | 42 | myco_tube_tkk-01-0018.1.scaffolds | 100 | 99 |
| 15 | mycobacterium_tuberculosis_md17888.0.scaffolds | 100 | 99 | 43 | myco_tube_tkk-01-0022.1.scaffolds | 100 | 99 |
| 16 | mycobacterium_tuberculosis_md18090.0.scaffolds | 100 | 99 | 44 | myco_tube_tkk-01-0027.1.scaffolds | 83 | 99 |
| Romania  "TB-ARC Romania initiative, Broad Institute (broadinstitute.org)" | | | | 45 | myco_tube_tkk-01-0037.1.scaffolds | 100 | 99 |
|  | name | Query cover | ident | 46 | myco_tube_tkk-01-0039.1.scaffolds | 85 | 99 |
| 1 | mycobacterium_tuberculosis_2074cj.0.scaffolds | 100 | 99 | 47 | myco_tube_tkk-01-0042.1.scaffolds | 100 | 99 |
| 2 | mycobacterium_tuberculosis_2097hd.0.scaffolds | 100 | 100 | 48 | myco_tube_tkk-01-0048.1.scaffolds | 100 | 99 |
| 3 | mycobacterium_tuberculosis_2099hd.0.scaffolds | 100 | 99 | 49 | myco_tube_tkk-01-0049.1.scaffolds | 100 | 99 |
| 4 | mycobacterium_tuberculosis_2100hd.0.scaffolds | 100 | 100 | 50 | myco_tube_tkk-01-0050.1.scaffolds | 100 | 99 |
| 5 | mycobacterium_tuberculosis_2230bh.0.scaffolds | 100 | 100 | 51 | myco_tube_tkk-01-0084.1.scaffolds | 100 | 99 |
| 6 | mycobacterium_tuberculosis_2231bh.0.scaffolds | 100 | 100 | 52 | myco_tube_tkk-01-0087.1.scaffolds | 100 | 99 |
| 7 | mycobacterium_tuberculosis_2483ar.0.scaffolds | 100 | 100 | 53 | myco_tube_tkk-01-0088.1.scaffolds | 85 | 99 |
| 8 | mycobacterium_tuberculosis_2485ar.0.scaffolds | 100 | 100 | 54 | myco_tube_tkk-01-0089.1.scaffolds | 100 | 99 |
| 9 | mycobacterium_tuberculosis_3280cj.0.scaffolds | 100 | 100 | 55 | myco_tube_tkk-01-0090.1.scaffolds | 100 | 99 |
|  |  |  |  | 56 | myco_tube_tkk-01-0093.1.scaffolds | 85 | 99 |


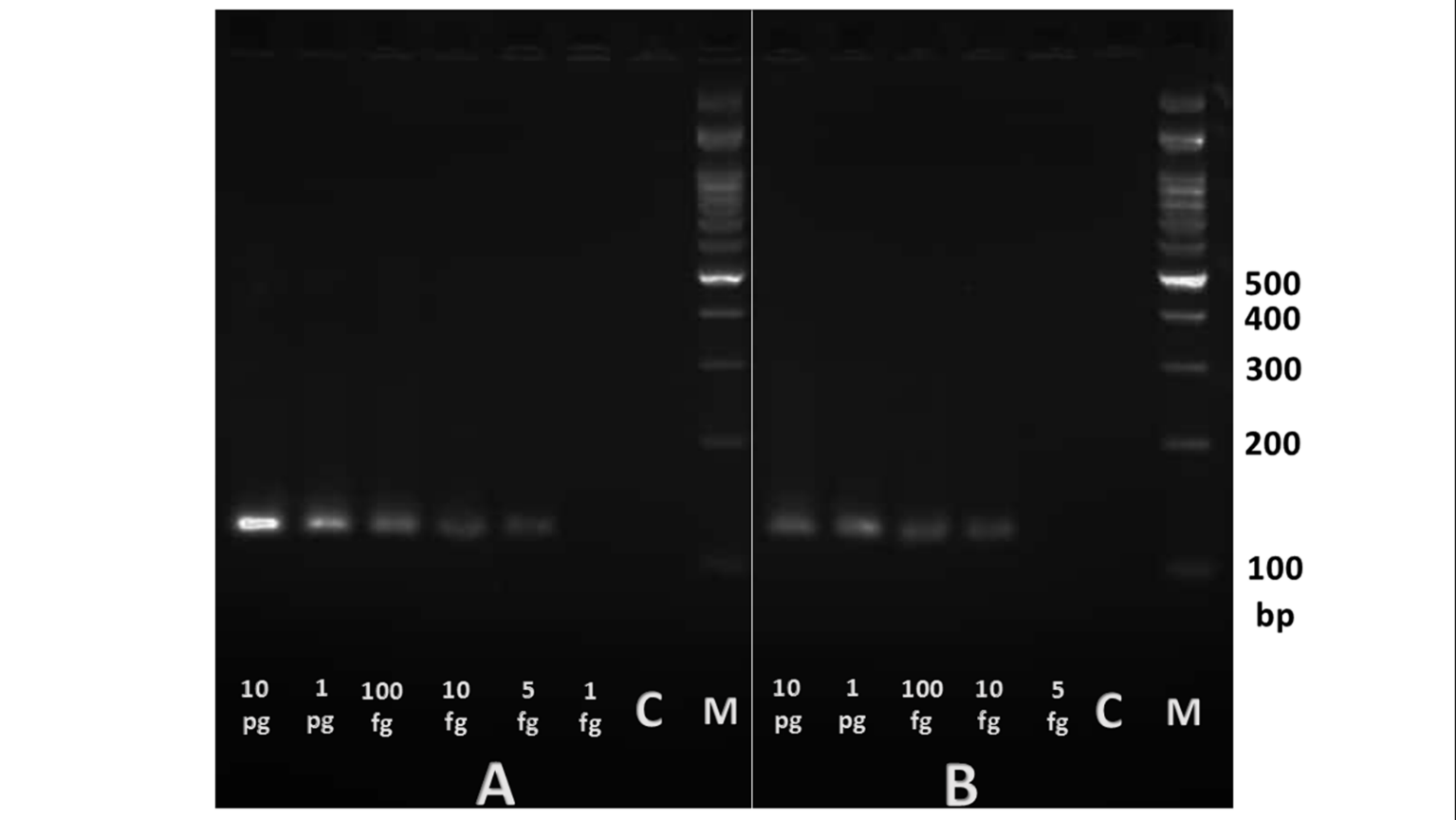


**Figure S 1.** Gel electrophoresis of 5KST-PCR products. It shows target amplicons at different concentrations of M. tuberculosis H37Rv genomic DNA spiked in processed clinical specimen as the template. 1 µl of sample for the group A and 2 µl of sample for the group B were used for the 5KST-PCR test. The final amount of DNA used in both 5KST-PCR groups was equal and the only difference was the amount of clinical specimen used in the reactions. The amount of template DNA used in each reaction is shown on the figure. M: 100 bp DNA size marker, C: Negative control
